# A call to action to advance global research priorities related to Undetectable = Untransmittable

**DOI:** 10.1002/jia2.70069

**Published:** 2025-12-18

**Authors:** Sarah K. Calabrese, Jacob Bor, Mandisa Dukashe, David A. Kalwicz, Kennedy Mupeli, Dorina Onoya, Lucy Stackpool‐Moore, Bruce Richman

**Affiliations:** ^1^ Department of Psychological and Brain Sciences George Washington University Washington DC USA; ^2^ Department of Prevention and Community Health George Washington University Washington DC USA; ^3^ Department of Global Health Boston University Boston Massachusetts USA; ^4^ Health Economics and Epidemiology Research Office University of Witwatersrand Johannesburg South Africa; ^5^ South African National AIDS Council Pretoria South Africa; ^6^ Centre for Youth of Hope Gaborone Botswana; ^7^ Watipa Sydney New South Wales Australia; ^8^ ASHM Sydney New South Wales Australia; ^9^ Prevention Access Campaign New York New York USA

**Keywords:** HIV, key and vulnerable populations, public health, treatment, Treatment as Prevention, Undetectable = Untransmittable

## Abstract

**Introduction:**

The Undetectable = Untransmittable (U = U) message has failed to reach many people living with HIV (PLHIV) and their communities despite evidence of its favourable impacts.

**Discussion:**

We describe and contextualize six global research priorities related to U = U, which focus on: (1) examining the effects of U = U messaging on health and economic outcomes; (2) illuminating and addressing barriers to U = U communication among healthcare providers, policymakers and other stakeholders; (3) expanding U = U research to include all key populations disproportionately affected by HIV; (4) addressing limited and inequitable access to information and resources; (5) determining how to optimally communicate about U = U in the context of evolving scientific knowledge and guidelines; and (6) collaborating on parallel studies across countries to improve comparability of study findings and identify cross‐cultural differences.

**Conclusions:**

Future research targeting these six priorities is needed to guide effective messaging about U = U in healthcare settings and public health programmes throughout the world. Ultimately, bridging existing gaps in U = U awareness, understanding and acceptance can enable PLHIV and others to reap the benefits associated with this valuable message.

## 1 INTRODUCTION

Decades of scientific evidence demonstrating the preventive benefits of antiretroviral therapy (ART) have contributed to sweeping changes in HIV policy all over the world, broadening HIV treatment eligibility and informing targets for HIV diagnosis, linkage to care and viral suppression. However, the specific conclusion that people living with HIV (PLHIV) whose viral load is consistently suppressed to an undetectable level cannot sexually transmit HIV [1, 2, 3, 4], commonly referred to as “Undetectable = Untransmittable” (“U = U” [5]), has not been effectively communicated to PLHIV and their communities [6, 7, 8, 9, 10, 11, 12].

The U = U campaign, a community‐led movement to raise global awareness about U = U, was launched in 2016 based on several large‐scale studies demonstrating zero occurrences of HIV transmission between serodifferent partners when the partner living with HIV maintained an undetectable viral load (e.g. HPTN052, PARTNER1 [2, 4]). Medical and scientific consensus grew in the years that followed [13], bolstered by corroborating evidence from the Opposites Attract and PARTNER2 studies [1, 3], announced in 2017–2018 [14, 15]. Yet, scepticism that ART eliminates sexual HIV transmission risk has persisted among PLHIV and others [16, 17, 18, 19, 20, 21].

U = U awareness, understanding, acceptance and messaging have been linked to numerous psychosocial and other health‐related benefits (see Table 1 [21, 22, 23, 24, 25, 26, 27, 28, 29, 30, 31, 32]). The U = U message also aligns with the larger public health imperative to end HIV transmission worldwide [33]. Therefore, research is needed to address existing barriers to effective U = U messaging in healthcare settings and public health programmes.

The suspension of US foreign aid, including the President's Emergency Plan for AIDS Relief (PEPFAR), in January of 2025, along with anticipated changes to the international funding landscape in the coming years, have and will continue to drastically disrupt HIV prevention and treatment service delivery [34, 35, 36]. In those countries with continued access to ART, U = U messaging could help to maximize its concurrent preventive and therapeutic benefits.

**Table 1:** Psychosocial and other health‐related benefits linked to U = U

Benefits	Research examples^a^	
Stigma reduction	Quantitative survey of 3070 members of the general public in Hong Kong (conducted in 2023) found that U = U unawareness and scepticism were associated with higher odds of expressing a pattern of modest, moderate or high (vs. low) HIV stigma‐related attitudes	Lau et al. [21]
	Quantitative survey of 30,361 GBOMSM living with HIV in the United States (conducted in 2018–2019) found that 79% thought U = U would favourably impact stigma and 82% reported U = U made them feel better about their HIV status (reduced self‐stigma)	Rendina et al. [22]
	Quantitative survey of 1083 PLHIV in Canada (conducted in 2018–2024) found that most of those who had heard of U = U (*n* = 780) reported that U = U had affected their lives positively (60%), would reduce stigma (80%), would effectively change public opinion (92%) and would change the opinions of individuals who were the most judgemental about PLHIV (64%)	Tran et al. [23]
Greater sexual openness, pleasure and intimacy	In‐depth qualitative interviews with nine young GBOMSM in Brazil (conducted in 2019–2021) found that having an undetectable viral load contributed to a sense of freedom to explore sexual pleasure	Duarte et al. [24]
	In‐depth qualitative interviews, repeated annually, with 17 current or former PrEP‐using GBOMSM in Canada (conducted in 2020–2022) found that some men became more open to having sex with PLHIV as their confidence in U = U increased over time	Grace et al. [25]
	In‐depth qualitative interviews with 43 PLHIV in Thailand (conducted in 2022) found that U = U made them “brave enough” to engage in sexual/romantic relationships and love others	Phanuphak et al. [26]
Alleviation of worry, anxiety, shame and guilt	Quantitative survey of 820 PLHIV in Australia (conducted in 2018) found that those who were not aware of U = U had higher odds of fearing onward HIV transmission	Norman et al. [27]
	In‐depth qualitative interviews with 27 PLHIV and 24 HIV‐negative people reporting current or past serodifferent relationships in Canada (conducted in 2016–2019) found that knowing U = U alleviated fears for mixed‐status couples and brought them a sense of comfort and safety	Ryan et al. [28]
	In‐depth qualitative interviews with 27 PLHIV and three focus group discussions (*n* = 27) with PLHIV in South Africa (conducted in 2021) found that those who believed in U = U expressed less anxiety around HIV transmission, the sense of a weight being lifted and the perception that they could be “fully moral actors”	Sineke et al. [29]
Better health and HIV service engagement	Randomized trial with 135 PLHIV in South Africa (conducted in 2022–2023) found that those who received U = U messaging via an app and monthly text messages in addition to standard HIV counselling (vs. only standard HIV counselling) had higher odds of retention in care and, in the unadjusted analysis, higher odds of documented viral suppression	Bor et al. [30]
	Quantitative survey of 2389 PLHIV from 25 countries (conducted in 2019–2020) found that those who were aware of U = U had higher odds of self‐reported viral suppression and optimal physical, mental, and overall health	Okoli et al. [31]
	Cluster randomized trial with 1048 HIV‐negative/status‐unknown men in South Africa (conducted in 2020) found that those who received a peer‐delivered invitation for HIV testing that incorporated U = U messaging (vs. an invitation without U = U messaging) had higher odds of subsequently testing for HIV	Smith et al. [32]

Abbreviations: GBOMSM, gay, bisexual and other men who have sex with men; PLHIV, people living with HIV.

^a^ Examples are included for illustrative purposes and are not intended to represent a comprehensive review of the literature.

In this Commentary, we describe and contextualize six research priorities that emerged from a multistakeholder, international research forum that we convened in 2024 to establish global priorities for research related to U = U and its translation to programmatic delivery (link). We conclude with our perspective on the advancement of U = U‐related research, highlighting the need for funding and other resources to propel future progress.

## 2 DISCUSSION

### 2.1 Priority 1. To examine the effects of U = U messaging on health and economic outcomes

Much of the published research pertaining to U = U messaging has concentrated on U = U awareness, understanding and/or acceptance [16, 18, 19, 37], as well as associated implications for psychosocial wellbeing and sexual decision‐making [22, 23, 26, 38, 39, 40, 41] among PLHIV and other community members. These lines of inquiry are valuable and could be expanded to encompass other mental health, quality of life and partner‐related outcomes. However, some target audiences, including healthcare providers and public health officials, may be more responsive to or compelled by evidence of favourable clinical/public health and economic effects, which is presently sparse. Additionally, whereas much of the literature to date relies on qualitative and cross‐sectional data, study designs incorporating randomization to messaging conditions and longitudinal follow‐up could strengthen inferences about causality.

Clinical/public health outcomes of primary interest include individual‐level HIV service engagement (e.g. HIV testing, linkage to and retention in care), ART initiation and adherence, and viral suppression, in addition to population‐level HIV incidence and viral suppression metrics. Monitoring for adverse sexual health effects (e.g. increased rates of other sexually transmitted infections) could clarify the extent of such effects and the value of integrating strategies to mitigate them into U = U messaging initiatives.

Although several randomized clinical trials involving messaging about ART reducing (rather than eliminating) HIV transmission risk have shown favourable impacts on clinical/public health outcomes [42, 43], to our knowledge, there have been only two randomized clinical trials assessing the impact of *U = U* messaging on clinical/public health outcomes (e.g. HIV testing, retention in care), both of which were conducted in South Africa and yielded promising results (see Table 1 [30, 32]). Additional intervention studies in other settings could bolster current evidence and offer insights about how best to deliver U = U messaging in different contexts.

Economic outcomes of interest include cost savings and cost‐effectiveness. Future research could consider the cost of implementing U = U messaging initiatives relative to the number of people who consequently avoid HIV acquisition, and relative to associated cost savings. Modelling could also help to inform investments in U = U messaging relative to pre‐exposure prophylaxis (PrEP) coverage, given the potential offsetting effects of treatment and PrEP with respect to preventive gains. That is, the gains associated with investing in U = U messaging may be greater in settings where PrEP coverage is lower, if ART remains accessible. Additionally, cost‐effectiveness studies could examine the cost of implementing U = U messaging initiatives that promote ART adherence and retention in care relative to the costs associated with drug resistance resulting from suboptimal adherence and regimen discontinuation, and relative to the costs of “reactively” locating and reengaging PLHIV who have disengaged from care.

Past modelling research has established the cost‐effectiveness of Treatment as Prevention (TasP), or the prevention of onward HIV transmission via viral suppression with ART. The concept of TasP underlies and predates U = U, originating at a time when ART was recognized to reduce, but not necessarily eliminate, sexual transmission risk. Such modelling research has shown that early initiation of ART (vs. delaying initiation until further disease progression) can be economically advantageous even when residual transmission risk at virologically suppressed levels is assumed, in part because of the savings associated with fewer people acquiring HIV [44]. However, we are unaware of any published studies that have rigorously examined the cost‐effectiveness of U = U messaging.

Future randomized trials and modelling studies assessing the impacts of U = U messaging may confront challenges, such as “contamination” of the standard‐of‐care arm in messaging trials. In healthcare settings where existing evidence has already sufficiently motivated U = U message delivery, implementation research, including that which addresses communication barriers (Priority 2), may be a more pressing need.

### 2.2 Priority 2. To illuminate and address barriers to U = U communication among healthcare providers, policymakers and others who are integral to U = U message delivery

Providers and other stakeholders need to be informed about U = U and be willing to relay the U = U message to PLHIV and others. However, previous studies have documented hesitancy among some providers [6, 7, 8, 9, 26, 45]. Reasons for such hesitancy include lack of understanding or disbelief regarding the underlying science of U = U, corresponding discomfort with the absolute nature of the message (e.g. “zero risk”) and perceived legal or moral liability if transmission were to occur. Hesitancy also stems from worry about viral load fluctuations causing periods of transmissibility and, likewise, unease about the low frequency of routine viral load testing to confirm that patients’ viral loads remain undetectable. Additionally, providers have expressed concerns about the potential impact of the U = U message on patient behaviour (e.g. increased condomless sex) and health (e.g. other STIs), as well as doubts about the relevance of the message to their patients [6, 7, 8, 9, 26, 45].

Among providers and other stakeholders willing to communicate about U = U, well‐meaning efforts to convey the U = U message can be undermined by use of language that is ambiguous (e.g. “virtually impossible”) or suggestive of enduring risk, however, minuscule (e.g. “extremely low,” “negligible” [46, 47]). Stigma may also interfere with providers’ communication about U = U, even without their knowing [48]. Limits on patient visit time dictated by health centre policies, and providers’ deprioritization of U = U relative to other health topics according to the perceived needs and preferences of their patients, can further inhibit U = U communication [6].

Research is needed to develop and evaluate interventions that address these and other emergent communication barriers and could be conducted as part of larger implementation studies. Such interventions could include educating providers about U = U and training them to accurately counsel patients, potentially drawing upon established curricula or guidelines [26, 49, 50]. Interventions conducted with providers and other stakeholders could be evaluated with respect to both direct outcomes (e.g. perceived confidence delivering the U = U message) as well as the indirect health and economic outcomes described in Priority 1. Research targeting areas of uncertainty that providers and other stakeholders have identified as contributing to hesitancy would yield valuable insights that could be integrated into educational interventions. For example, studies examining the risk of viral rebound at different testing intervals could reassure providers that an undetectable viral load is likely to be sustained between tests, particularly in settings where testing is performed annually rather than every 6 months.

### 2.3 Priority 3. To expand research on U = U messaging to include all key populations disproportionately affected by HIV

Much of the published research on U = U messaging has concentrated on gay, bisexual and other men who have sex with men (GBOMSM). Such research has largely been based in high‐income countries, where this population accounts for a large proportion of people who newly acquire HIV [19, 22, 25], and is vital to continue, particularly with subgroups facing disproportionately high rates of acquisition, such as Black GBOMSM in the United States [51]. However, comparable research among other populations living with or at elevated risk for HIV is needed, given that findings among GBOMSM cannot be assumed to generalize to other groups. Nearly half of the 1.3 million people who newly acquire HIV annually and 65% of the 39.9 million people currently living with HIV reside in sub‐Saharan Africa [52], where the predominant mode of transmission is heterosexual sex, and most of the people who newly acquire HIV are women and girls [53, 54]. It would be valuable to focus on women and girls—and their male partners—along with other under‐researched populations disproportionately affected by HIV (e.g. trans and gender diverse people, sex workers), in future U = U messaging research and to design studies and messaging intervention in partnership with these groups.

### 2.4 Priority 4. To address limited and inequitable access to HIV information and resources

There is a profound global information injustice regarding access to the scientific evidence on U = U. Information about U = U has not been equitably disseminated across populations affected by HIV. A 2021 literature review determined that awareness of U = U or TasP was “widespread” among GBOMSM who are living with HIV in high‐income countries but lagged considerably among populations throughout sub‐Saharan Africa and elsewhere in the world [12]. Disparities exist not only with respect to access to U = U information but also the medical resources needed to establish and maintain an undetectable viral load, including medication, services and laboratory equipment. These disparities are further reflected in the variable success that countries have achieved thus far in meeting diagnosis, treatment and viral suppression targets set by the United Nations Joint Programme on HIV/AIDS [52]. PLHIV who are unable to access treatment and, consequently, incapable of suppressing their viral load to an undetectable level suffer the physical health consequences of uncontrolled viraemia and are denied the opportunity to reap the psychosocial benefits associated with U = U. Addressing inequities in access to resources is largely contingent on funding and infrastructure, and research can help to elucidate the impact and corresponding needs created by recent international aid disruptions and to inform remediation strategies.

### 2.5 Priority 5. To determine how to optimally communicate about U = U in the context of evolving scientific knowledge and guidelines

Scientific advancements generate new HIV risk‐related information that needs to be communicated effectively to potential beneficiaries. For example, a 2023 systematic review concluded that the risk of sexually transmitting HIV with low‐level HIV viraemia (200−999 copies/ml) was “almost zero” [55]. The review coincided with a policy brief released by the World Health Organization, which described three viral load categories—unsuppressed (>1000 copies/ml), suppressed (detected but ≤1000 copies/ml) and undetectable (not detected by test used)—and encouraged new messaging about “almost zero or negligible risk” of sexual transmission for PLHIV in the suppressed category [56]. Because “zero risk” is a crucial element of the U = U message, this new viral load categorization and messaging about “almost zero risk” could be a source of confusion. The two messages (zero risk at undetectable levels, almost zero risk at detected but suppressed levels) are not contradictory but could be perceived as such if the nuanced distinction between undetectable and suppressed is not fully understood by message recipients. Thus, research is needed to determine how to effectively communicate this finding and other emergent scientific findings related to HIV risk to potential beneficiaries without undermining the U = U message. Likewise, the potential efficacy of combining U = U messaging with other risk or preventive messaging, such as messaging about HIV vertical transmission risk or prevention of other sexually transmitted infections, merits further investigation.

### 2.6 Priority 6. To collaborate on parallel studies across multiple countries simultaneously to improve comparability of study findings and identify cross‐cultural differences

Most published studies examining U = U awareness, understanding, acceptance or messaging implications have been conducted within a single country or community (see Okoli et al. [31] for an exception). However, collaboration to codesign and execute a multinational study or to simultaneously execute parallel studies across multiple countries offers numerous advantages. Rather than each research group independently developing protocols, measures and other study materials, codeveloping a single set of materials or adapting one another's could accelerate study execution. Furthermore, the uniformity in study materials and simultaneity of study execution would allow for more rigorous comparisons of results to be drawn across countries. Conducting studies in multiple countries with parallel measures could illuminate important similarities and variations in U = U knowledge, attitudes or impact, which could be informative to policymakers seeking evidence that a given intervention will be effective in their own country or community.

## 3 CONCLUSIONS

The six identified research priorities highlight existing gaps in scientific evidence. Addressing these gaps could strengthen support for U = U dissemination and equip stakeholders with evidence‐based dissemination strategies.

We advance these priorities while also acknowledging our positionality. The conceptualization of the priorities and planning of the global research forum that informed them were led by an academic researcher who is a White heterosexual cisgender woman from the United States. Although our author group and forum attendees were diverse with respect to professional background, race, sexual orientation, gender, nationality and HIV status, multiple voices were not represented, and we do not assume that these global priorities are shared by all.

To translate the identified priorities into research action, efforts are needed to motivate researchers to design and conduct studies. To this end, funding is paramount. The termination of thousands of research grants, collectively worth billions of US dollars, by the National Institutes of Health and other US federal agencies beginning in early 2025 has introduced major setbacks worldwide, fostering an immediate need to advocate for US funding recommitments and increase research investments from alternate sources [57, 58, 59, 60]. Beyond funding, endorsement of the identified U = U research priorities by institutional and individual leaders in the field of HIV prevention and treatment may elevate their significance. Additionally, cultivating ongoing partnerships with local community members and organizations would facilitate the conceptualization and execution of studies that address these priorities while maintaining congruency with community needs and goals. Collectively, these motivational strategies could galvanize research addressing the identified priorities, strengthening the evidence to inform future U = U messaging initiatives and ultimately amplifying global impact.

## AUTHORS’ CONTRIBUTIONS

SKC drafted and revised the commentary. All other co‐authors (JB, MD, DAK, KM, DO, LS‐M and BR) reviewed and provided feedback on the initial draft and approved the final version. DAK checked all literature citations and references.

## COMPETING INTERESTS

The author group includes members of the planning committee for the research forum referenced in the Introduction. SKC, LS‐M and BR received indirect compensation and support for travel expenses from Gilead Sciences and ViiV Healthcare for planning and coordinating the research forum. BR is the founder of the U = U movement and Prevention Access Campaign, the website for which is cited in connection to U = U. MD, KM and BR contributed to and/or endorsed resources referenced in our discussion of Priority 2. The authors have no other competing interests to disclose.

## FUNDING

The authors did not receive any funding to support the preparation of this Commentary.

## Data Availability

Data sharing is not applicable to this article as no datasets were generated or analysed during the current study.
